# Impact of SARS-CoV-2 virus pandemic on the future of cadaveric dissection anatomical teaching

**DOI:** 10.1080/10872981.2020.1823089

**Published:** 2020-09-15

**Authors:** Setthasorn Zhi Yang Ooi, Rucira Ooi

**Affiliations:** a Cardiff University School of Medicine, University Hospital of Wales Main Building, Heath Park, Cardiff, United Kingdom; b Department of Obstetrics and Gynaecology, Princess of Wales Hospital, Bridgend, United Kingdom

**Keywords:** Undergraduate, anatomy teaching, cadaver, dissection, Coronavirus

## Abstract

The SARS-CoV-2 virus pandemic has left a huge impact on medical education globally. An area that has not been discussed in medical education is the potential implications of the cessation of body and organ donations on medical education. We explore the implications of this on the future of cadaveric dissections in anatomy teaching amidst the SARS-CoV-2 virus pandemic.

## Letter

The SARS-CoV-2 virus pandemic has left a huge impact on medical education globally. An area that has not been discussed in medical education is the potential effects of the cessation of body and organ donations on medical education. We explore the implications of this on the future of cadaveric dissections in anatomy teaching amidst the SARS-CoV-2 virus pandemic.

The recent release of guidance from the British Medical Association (BMA) and the British Government stated that suspected or confirmed Coronavirus patients were advised to undergo cremation after death [1,2]. This practice is advised due to the lack of evidence on the risk of transmission of the virus from the deceased to the living [1,2]. Although this practice is not compulsory, family members who choose against cremation have to follow strict guidelines on the care and transportation of the deceased; this includes the use of Personal Protective Equipment (PPE) and the practice of social distancing from the deceased. The Human Tissue Authority has also released a statement that medical schools in the UK (UK) are not allowed to receive body donations due to the SARS-CoV-2 virus outbreak [3]. These factors may lead to a decrease in body and organ donations, which may subsequently affect the delivery of anatomical teaching through cadaveric dissections in the future.

In the upcoming academic years, incoming students of medical schools who practice cadaveric dissection teaching will miss out on the opportunity to learn anatomy through dissections. This letter aims to explore the implications of the cancellation of cadaveric dissection in anatomy teaching as a result of the SARS-CoV-2 virus pandemic.

What does this mean for medical students at schools who traditionally teach anatomy through cadaveric dissections?

1. Students will miss out on the opportunity to learn and develop professional skills and values that will be important to them as future doctors. As cadavers are often known to be the first ‘silent teacher’ to medical students, this is usually the first encounter for students to understand and appreciate the importance of medical professionalism, ethics, and confidentiality. Cadaveric dissections also teach students to show their respect and gratitude to the generosity of the body donors and their families.
2. Students will not have the early exposure to develop skills of handling basic surgical instruments and to develop manual dexterity. Cadaveric dissections usually allow students to learn the skill of using a scalpel, various types of scissors (namely curved, straight, and Metzenbaum), and various types of forceps (namely blunt, fine, and rat-toothed).
3. Students may not be able to appreciate the three-dimensional relationship of anatomical structures as much as they would through cadaveric dissections. Cadaveric dissections offer a full hands-on experience in learning anatomy.
4. Students will miss out on the opportunity to develop and enhance their teamwork skills. It often requires the effort of a team of students to dissect through a cadaver.

Although there is a current setback in anatomical teaching through cadaveric dissection caused by the cessation of organ and body donations, we are still fortunate enough to have other platforms to compensate for this. Medical schools that practice cadaveric dissections can approach anatomical teaching through different methods, namely using prosections, body art, virtual reality, or plastic models. The benefits of these in teaching anatomy in modern medical curricula has been supported by published evidence [4].

However, as medical schools resume to a ‘new normal’ of in-person teaching once the pandemic resolves, it would be heartbreaking to see anatomical teaching through cadaveric dissections being completely replaced as a result of this pandemic. Medical schools should recognize that there are invaluable morals and qualities that only a ‘silent teacher’ can speak to a student.

‘Mortui vivos docent.’
